# *Tetratrichomonas gallinarum* granuloma disease in a flock of free range layers

**DOI:** 10.1080/01652176.2019.1682714

**Published:** 2019-10-18

**Authors:** W. J. M. Landman, N. Gantois, J. H. H. van Eck, H. M. J. F. van der Heijden, E. Viscogliosi

**Affiliations:** a GD Animal Health, Deventer, the Netherlands;; b CNRS, Inserm, CHU Lille, Institut Pasteur de Lille, U1019 – UMR 8204 – CIIL – Centre d’Infection et d’Immunité de Lille, University of Lille, Lille, France;; c Department of Farm Animal Health, Faculty of Veterinary Medicine, Utrecht University, Utrecht, the Netherlands

**Keywords:** Layer chickens, ducks, *Tetratrichomonas gallinarum*, *Simplicimonas* sp., *Histomonas meleagridis*, granulomas, *In situ* hybridization, nested PCR

## Abstract

Granuloma disease in a flock of free range productive layers in the Netherlands in 2017 is described. The disease resembled granuloma outbreaks in layers caused by *Tetratrichomonas gallinarum* in 2013 and occurred in the same area in which the rearing farm considered as the source of the 2013 outbreaks was located. Between 55 and 84 weeks of age mortality was 20.3% (breeder’s norm 3.9%). All dead hens examined (*n* = 20) showed granulomas especially in liver and ceca. Nine hens with or without liver and/or ceca granulomas were examined for trichomonads in mentioned organs by *in situ* hybridization (ISH), nested PCR, and cloning and sequencing. Ceca were also examined by culture. *T. gallinarum* ISH was positive in all livers and ceca with granulomas and negative in case granulomas were absent. *T. gallinarum* strain 13/16632, which caused the 2013 outbreaks was found in 4/8 hens with granulomas. Moreover, other trichomonads were detected: a *T. gallinarum* strain GPO-like and a *Simplicimonas* sp. strain GABC1-like. Mixed infections also occurred. Infectious causes of granuloma disease other than the afore-mentioned trichomonads could be excluded. Trichomonad DNA was not detected in environmental samples and wild ducks originating from the farm of concern, except for one duck in which the same *Simplicimonas* sp. as in hens was detected, leaving the source of the *T. gallinarum* infection in hens unknown. It is concluded that the herein described granuloma disease likely was caused by *T. gallinarum* strain 13/16632. However, the pathogenicity of the other trichomonads found remains to be clarified.

## 1. Introduction

Granulomas may develop when the immune system is not able to eliminate amongst others, disease inducing microorganisms resulting in chronic stimulation of the immune system. Lesions mainly consist of macrophages (histiocytes), epithelioid cells and multinucleated giant cells. Also other cells such as lymphocytes, plasma cells and fibroblasts may be present and necrotic/caseous debris is often found at the core of the granuloma (Williams and Williams 1983). A number of infectious causes of granulomas in chickens has been described, such as the trichomonad protozoa *Pentatrichomonas* (Allen 1936) later renamed to *Tetratrichomonas gallinarum* (Honigberg 1963) and *Histomonas meleagridis* (Hess and McDougald 2013), the fungus *Aspergillus fumigatus* (Arné et al. 2011) and the bacteria *Salmonella pullorum* (Shivaprasad 2000) and *Mycobacterium avium* (González et al. 2002). Moreover, certain *Escherichia coli* strains have been described as cause of a granuloma disease called coligranulomatosis that occurs sporadically in adult chickens, turkeys and partridges. The latter disease is not economically relevant as it affects only a few birds in a flock (Hjärre and Wramby 1945).

In 2013, severe granuloma disease outbreaks were observed in seven productive layer flocks housed on different farms in the Netherlands. Outbreaks were characterized by persistent increased mortality (3.7–11.0% exceeding the breeder’s norm in periods ranging from nine to 48 weeks, starting between 21 and 35 weeks of age) and by a high within flock incidence of granulomas. On average, 87% of birds from diseased flocks had granulomas (range 50–100% per flock) mainly in ceca and livers. Some flocks also showed decreased egg production and/or loss of mean egg weight (Landman et al. 2016). By fulfilling Koch’s postulates, it was shown that the outbreaks were caused by the trichomonad species *T. gallinarum* (Landman et al. 2016; Landman and Van Eck 2018). All affected flocks could be linked to one rearing farm, which seemed therefore to be the source of the *T. gallinarum* infection. However, no signs of disease had been observed at this rearing farm and it remained unknown how this farm became infected (Landman et al. 2016).

In the present case report, an outbreak of granuloma disease in 2017 closely resembling the 2013 outbreaks, is described in a flock of free range productive layers. Affected organs were examined for the presence of trichomonads (amongst others *T. gallinarum* and *H. meleagridis*) by DNA techniques (*in situ* hybridization (ISH), nested PCR and sequencing) and culture. Infection with tuberculosis bacteria was assessed by Ziehl–Neelsen staining of organ sections. Furthermore, granulomatous organs were examined histologically for the presence of bacteria and fungi.

Wild birds (ducks) captured on the affected farm and environmental samples taken at mentioned farm were screened in an attempt to elucidate the source of the infection in the laying hens.

## 2. Materials and methods

### 2.1. Farm and flock data

The farm studied in this report was surrounded by ditches and located in a wetland area with abundant wild waterfowl in the province Zeeland, the Netherlands. Its distance to the chicken rearing farm, which was linked to seven outbreaks of granuloma disease in laying hens caused by *T. gallinarum* in 2013 (Landman et al. 2016) was 3.8 km. In November 2016 at 19 weeks of age, the flock of concern was housed in a layer barn with litter as bedding. The flock consisted of 33,088 Dekalb White layer hens. The birds had been reared indoors in another house at the same farm. Until 39 weeks of age hens were kept indoors; thereafter they were given access to a free range area. Feed and water consumption were always normal. During the entire production period, egg production based on hens present exceeded the breeder’s norm: in the first half of the production period by 1.5–4%, in the second half by 5–10%. However, mean egg weight was permanently below the breeder’s norm, ranging from 0.5 to 3 g. Up to 55 weeks of age, mortality was very low: 0.85% of hens housed (breeder’s norm: 3.5%). From the afore-mentioned age, mortality increased rapidly and substantially. It persisted at a high level until the end of the production period at 84 weeks of age, resulting in 20.3% mortality of hens housed between 55 and 84 weeks of age (breeder’s norm: 3.9%). Hens were treated with tiamulin (Vetmulin^®^ 450 mg/g, Huvepharma, Antwerpen, Belgium) provided *via* the drinking water in a dose of 21 mg per kg body weight per day during 5 days, starting at the age of 61 weeks. During the treatment and thereafter hens were kept indoors.

### 2.2. Examination of layer hens, wild ducks and environmental samples

Hens were submitted to GD Animal Health, Deventer, the Netherlands (GD) twice: 8 fresh dead hens at 59 weeks of age and 24 hens (12 live hens and 12 fresh dead hens) one day before the tiamulin treatment started at 61 weeks of age. The latter hens were numbered individually in order to link the analysis results to single birds. Gross post-mortem examination was performed. Livers and ceca with or without macroscopic granulomas were examined histologically (haematoxylin and eosin (HE) and Ziehl–Neelsen stained sections) and by ISH for the trichomonad species *T. gallinarum* and *H. meleagridis*. Moreover, ceca with granulomas of a number of 61-week-old hens were used for the culture of trichomonads. In view of the high mortality, the hens first submitted to GD were also examined for avian influenza (AI) virus (AIV) infection and for the *E. coli* peritonitis syndrome (EPS) (Landman et al. 2013, 2014). Hereto, trachea and cloaca swabs (five pooled trachea and five pooled cloaca swabs) were examined by AI matrix gene real-time PCR, which detects all AI virus subtypes (Ward et al. 2004). Bacteriological analysis of femur bone marrow of four hens was performed to assess the presence of *E. coli* (Landman and Van Eck 2017b). Preparation of HE and Ziel–Neelsen stained tissue sections, culture of trichomonads from ceca in Diamond’s medium, and ISH for *T. gallinarum* and *H. meleagridis* were done as presented before (Landman et al. 2016; Landman and Van Eck 2018, Liebhart et al. 2006) with the difference that visualization of the probes for ISH was done using Magenta (GV925, EnVision FLEX HRP Magenta Substrate Chromogen System (Dako Omnis), Agilent Technologies Netherlands B.V., Amstelveen, the Netherlands). During a farm visit (hens were 61 weeks of age at that time), wild ducks (*Anas platyrhynchos*), Eurasian oystercatchers (*Haematopus ostralegus*), pigeons (different species) and European jackdaws (*Corvus monedula*) were seen on the premises. Five wild ducks were shot by a licensed hunter and sent to GD. Here, gross post-mortem examination was performed. Also, 12 environmental samples were taken during the farm visit: one water sample from a drainage channel parallel to the barn, one mud sample taken from a wet free range area between trees, two water samples from a ditch behind the barn, two water samples from a small water basin for sheep, one mud sample with waterfowl droppings taken from the soil of a sheep shelter, one water sample from a drainage channel running parallel to the back of the barn, two water samples from a large ditch running parallel to the front of the barn and two water samples from a rain basin at approximately 100 meters distance from the barn.

An overview of samples of 61-week-old hens examined and the test methods used is given in Table 1.

**Table 1.** Results of *T. gallinarum* ISH, nested PCR detection of trichomonads and identification by sequencing in 61-week-old layer hens with granuloma disease.

		Macroscopic granulomas^a^ in		ISH *T. gallinarum*		Nested PCR trichomonads, cloning and sequencing		
Hen no.	Live (L) or dead (D)	Liver	Ceca	Liver	Ceca	Liver	Ceca	Culture of protozoa from ceca
10	L	++	+	Positive	Positive	−	*Simplicimonas* sp.^b^	*Simplicimonas* sp.^b^ (44 h culture)
11	L	++	−^c^	Positive	Positive	−	*Simplicimonas* sp.^b^	*T. gallinarum*^d^ (44 and 92 h culture)
1	L	+++	+++	Dubious	Positive	*T. gallinarum*^e^	−	
2	L	−	+	Negative	Positive	−	−	
3	L	−	−	Negative		−	−	
4	D	+++	++	Positive	Positive	*T. gallinarum*^e^	*Simplicimonas* sp.^b^	
5	D	+++	++	Positive	Positive	−	−	*T. gallinarum*^e^ (44 and 92 h cultures)
16	D	++	++	Positive	Positive	−	−	
24	D	++	+++		Positive	−	*T. gallinarum*^e^ and *Simplicimonas* sp.^b^ (after cloning)	

ISH: *in situ* hybridization; +: one granuloma per hen, ++: some granulomas per hen and +++: many granulomas per hen; −: not detected; empty cell: not done.

a Macroscopic granulomas were always confirmed by histopathological analysis.

b 99% sequence identity with *Simplicimonas* sp. GABC1.

c In this case microscopic granulomas were observed.

d 99% sequence identity with *T. gallinarum* strain GPO.

e 100% sequence identity with *T. gallinarum* strain 13/16632.

### 2.3. DNA extraction of animal and environmental samples

DNA extracts were obtained from ceca and liver samples, and from cultures of these organs of 61-week-old hens, from ceca of wild ducks and from environmental samples.

DNA isolation was performed using a 10% suspension of liver and ceca samples in phosphate buffered saline (PBS). Hereto, 150 mg tissue was cut into a Magnalyser Green Beads tube (Roche Diagnostics, Almere, the Netherlands), adding the beads and 1.5 ml PBS. Tissues were homogenized in FastPrep-24 5 G Grinder disruption instrument (MPBio, Eschwege, Germany) during 3 min. The suspension was centrifuged at 13,000 ×*g* during 2 min and again during 1 min. The supernatants of both centrifugation steps were combined and 200 µl was used for DNA isolation.

An amount of 0.2 g of the environmental mud samples and of the sediments of the water samples, each to which 500 µl of PBS was added, were vortexed during 3 min and subsequently centrifuged during 1 min at 100 x*g*. DNA isolation was done with the MagMAX Pathogen RNA/DNA kit (ThermoFischer Scientific, Bleiswijk, the Netherlands) according to the instructions of the manufacturer, from 200 μl of the supernatants and of 50 μl of the cultures without prior tissue disruption treatment.

### 2.4. Detection and identification of trichomonads by DNA techniques

In order to identify trichomonad species, all DNA samples were screened by nested PCR, allowing the DNA amplification of the internal transcribed spacer 1 (ITS1)-5.8S rRNA-ITS2 region by use of trichomonad-specific primers. The first PCR was performed using the sense primer TRICHO-F (5′-CGGTAGGTGAACCTGCCGTT-3′) and the antisense primer TRICHO-R (5′-TGCTTCAGTTCAGCGGGTCT-3′) described by Jongwutiwes et al. (2000). The PCR was carried out for 40 cycles in a thermocycler (Bioer Technology, Life Eco, Hangzhou, China) with a 50 µl volume according to standard conditions for Platinum *Taq* high-fidelity DNA polymerase (InvitrogenTM, Life Technologies Europe BV, Bleiswijk, the Netherlands). Negative (samples without trichomonads) and positive (*Trichomonas vaginalis* DNA extracted from axenic culture) controls were included in the PCR series. The second amplification was performed with the sense primer TRICHO-FBIS (5′-GGTGAACCTGCCGTTGGATC-3′) and the antisense primer TRICHO-RBIS (5′-TCAGTTCAGCGGGTCTTCCT-3′) (Duboucher et al. 2006), using 1 µl of the first amplification as template and the same conditions as in the first PCR. Positive nested PCR products were purified and sequenced in both strands by Genoscreen (Lille, France). For one chicken sample, sequence chromatogram analysis revealed the presence of a double trace, suggesting mixed infection by at least two trichomonad isolates. Therefore, the corresponding nested PCR product was separated by agarose gel electrophoresis, and the band of the expected size (around 400 bp) was purified using the Wizard SV Gel and PCR clean-up system (Promega, Madison, USA). The purified PCR product was cloned in the T-vector, pCR 2.1-TOPO (Invitrogen) and amplified in *E. coli* One Shot TOP10 competent cells. Minipreparations of plasmid DNA were made using the NucleoSpin Plasmid kit (Macherey-Nagel, Düren, Germany). Five positive clones containing inserts of the expected size were selected arbitrarily and sequenced on both strands. The ITS1-5.8S rRNA-ITS2 sequences obtained in this study were deposited in GenBank under accession numbers MK496862 to MK496874. These sequences were aligned with each other and then compared with all of the ITS1-5.8S rRNA-ITS2 trichomonad sequences available from the National Center for Biotechnology Information (NCBI) server using the nucleotide Basic Local Alignment Search Tool (BLAST) program. Trichomonads were identified by determining the exact match or closest similarity to all known species and strains.

## 3. Results

Examination of the eight dead hens of 59 weeks of age (first submission to GD) revealed that all animals had degenerated ovaries and showed numerous granulomas in the liver. Six hens also possessed varying numbers of cecal granulomas. The granulomatous nature of the lesions was confirmed by histopathological analysis. Intestinal parasites were not detected. ISH showed the presence of *T. gallinarum* in the granulomas in one of two ceca and in two of three livers examined while *H. meleagridis* ISH was always negative (three livers and one cecum, both with granulomas were examined). AIV infection and EPS were excluded: AIV PCR of trachea and cloaca swabs was negative and bacteria were not isolated from bone marrow.

**Figure 1.**
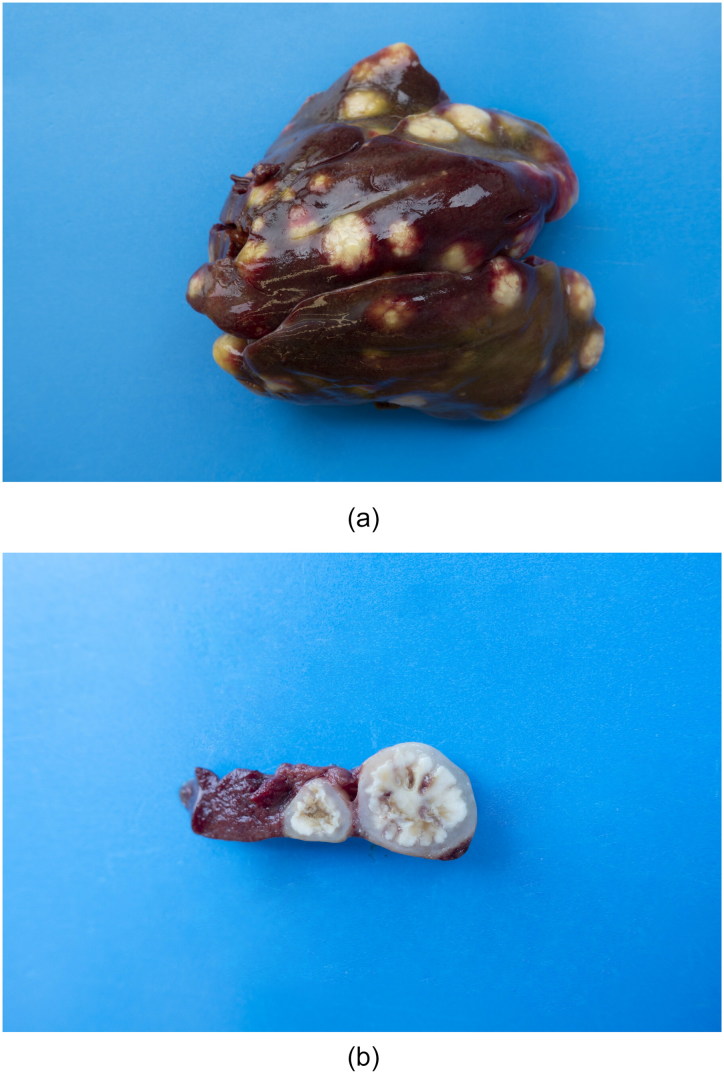
**(**a) Liver of a 61-week-old white layer with numerous large granulomas. (b) A transversally cut granuloma showing its cauliflower-like structure.

**Figure 2.**
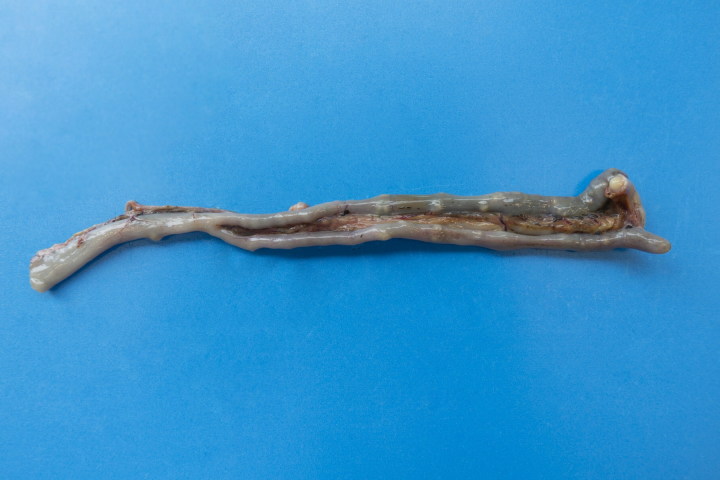
Cecum of a 61-week-old white layer with numerous small granulomas.

**Figure 3.**
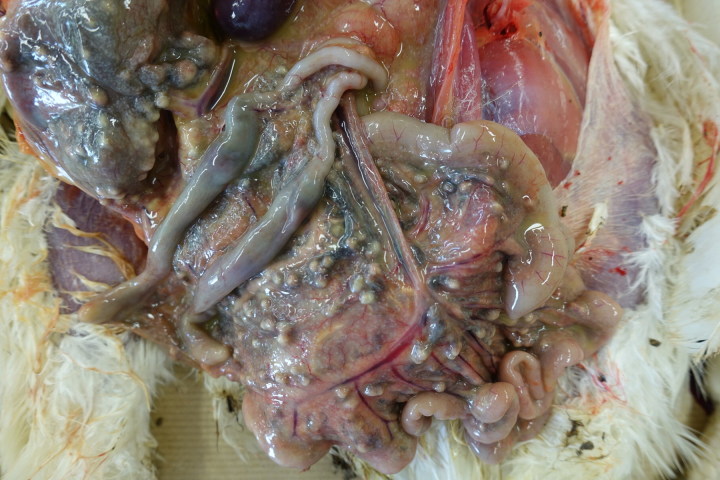
Mesenterium of a 61-week-old white layer with numerous small granulomas.

**Figure 4.**
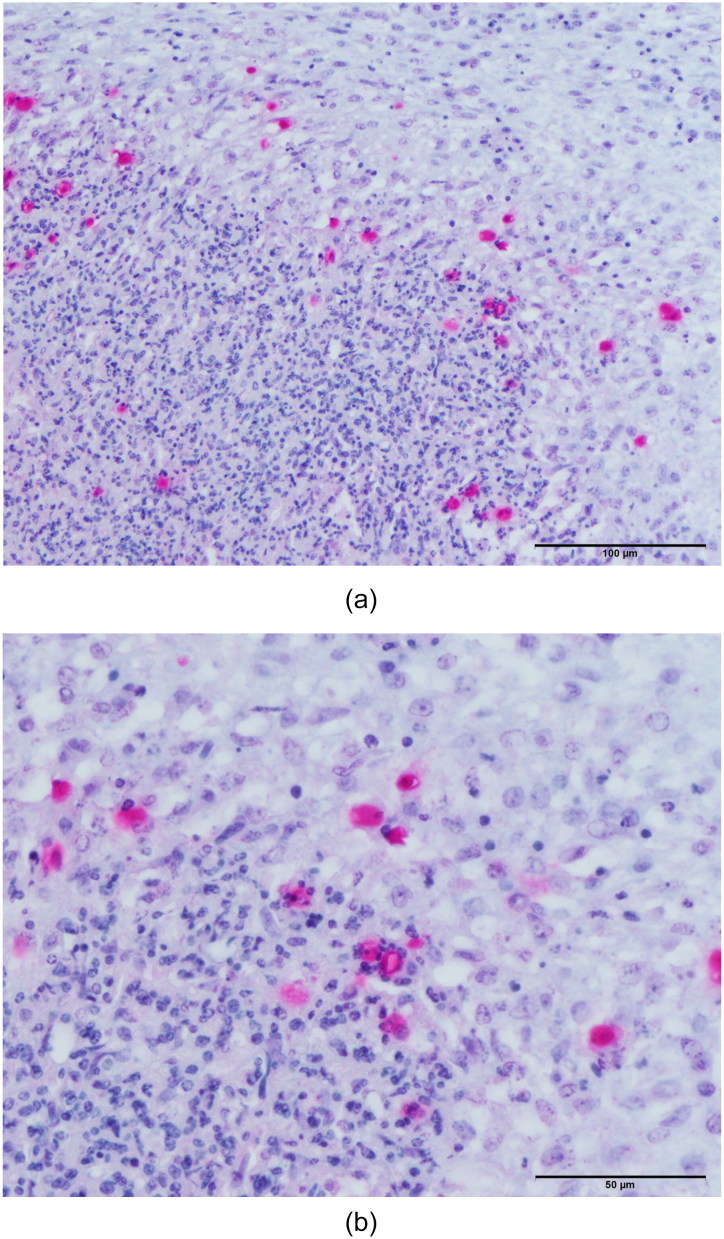
(a,b) *In situ* hybridization of a liver granuloma showing the presence of *T. gallinarum*. The section was counterstained with hematoxylin and eosin. Numerous parasites are predominantly present in the periphery of the granuloma.

**Figure 5.**
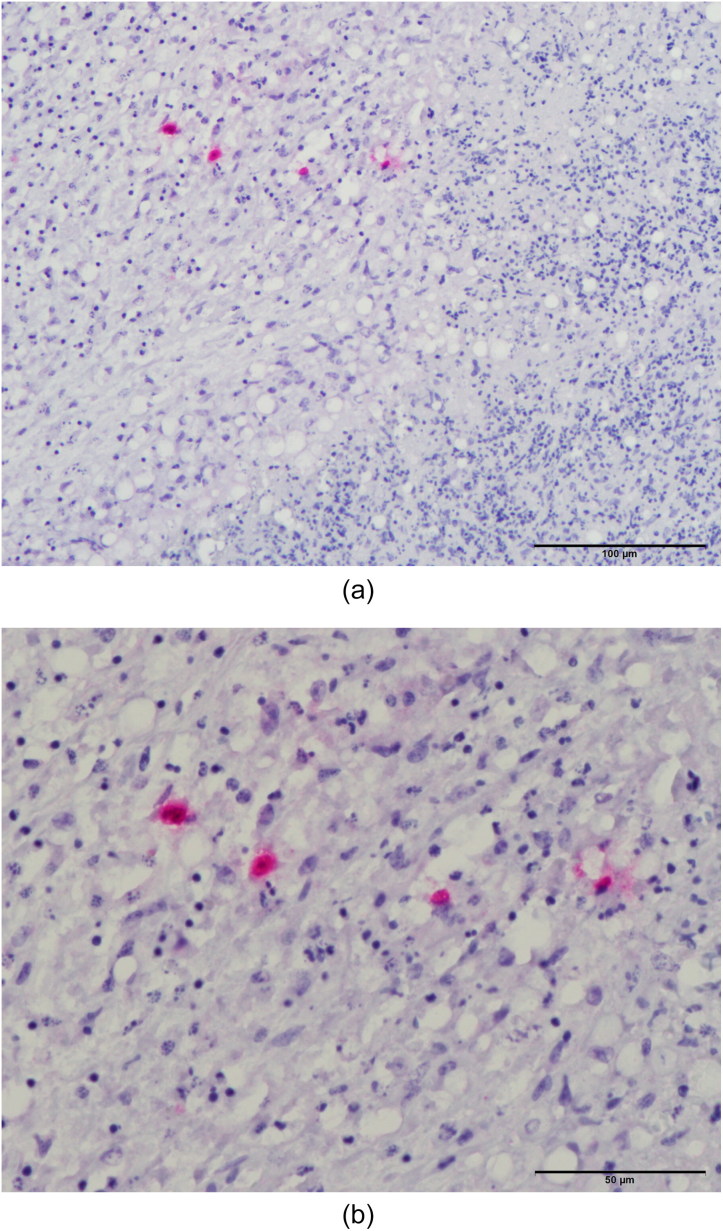
(a,b) *In situ* hybridization of a cecum granuloma showing the presence of four *T. gallinarum* parasites. The section was counterstained with hematoxylin and eosin.

Results of gross post-mortem examination of 61-week-old hens are presented in Table 2. All 12 dead hens showed numerous liver granulomas (Figure 1(a,b)) and nearly all had granulomas in the ceca (Figure 2). Furthermore, granulomas in spleen and mesenterium (Figure 3) were present in a number of birds (Table 2). Almost all of the dead hens had unproductive ovaries. Four of 12 live hens had some to numerous granulomas in liver and/or ceca and the majority of these hens had productive ovaries. Granulomas varied in size from 2 to 3 mm up to approximately 6–7 cm. Part of the results of further analysis of nine individually numbered hens (five hens submitted alive and four hens submitted dead) are given in Table 1. Except for one hen (Table 1, hen number 3), all hens showed macroscopic granulomas in liver and/or ceca. Their granulomatous nature was confirmed by histological analysis. Bacteria including acid fast types (Ziehl–Neelsen staining) and fungi were not observed in or around of any of the granulomas. Moreover, *H. meleagridis* ISH was negative in both, liver and cecum sections of all nine birds, while *T. gallinarum* ISH was positive in all livers (Figure 4(a,b)) and ceca (Figure 5(a,b)) with granulomas, and negative in case granulomas were not present (Table 1, hen numbers 2 and 3). *T. gallinarum* was found in the periphery of granulomas in numbers of 2–19 (mean 9) per granuloma in ceca (*n* = 10) and >100 per granuloma in livers (*n* = 5).

**Table 2.** Results of gross post-mortem examination of hens from a flock of 61-week-old white layers with granuloma disease.

		Number of hens with						
		Granulomas in liver	Granulomas in cecum	Productive ovary	Degenerated ovary	Inactive ovary	Reduced condition	Other findings
Twelve dead hens		12 (+++*)	10 (++ to +++)	1	7	4	7	5 × spleen granuloma (++); 1 × mesenterium granuloma (+++); 10 × swollen liver; 7 × pale kidneys
Twelve live hens	Five clinically depressed hens	1 (+++)	2 (+ and +++)	2	1	2	3	1 × perihepatitis; 1 x pale kidneys
	Seven clinically healthy hens	2 (++)	1 (+)	7	0	0	0	0

* +: one granuloma per hen, ++: some granulomas per hen; +++: many granulomas per hen.

The results of the detection and identification of trichomonads by DNA techniques in animal samples are presented in Table 1. A *T. gallinarum* isolate presenting 100% sequence identity with the homologous sequence of the strain 13/16632 (GenBank accession number LK031731) causing the outbreaks of 2013 (Landman et al. 2016) was found in livers and/or ceca of four hens with granulomas (Table 1, hen numbers 1, 4, 5 and 24). In protozoal culture of ceca from another hen with granulomas (Table 1, hen number 11), a *T. gallinarum* isolate presenting 99% sequence identity with that of *T. gallinarum* strain GPO isolated from *Gallus gallus* (GenBank accession number AY245129) was identified. Moreover, a *Simplicimonas* sp. isolate exhibiting 99% sequence identity with the homologous sequence of *Simplicimonas* sp. strain GABC1 isolated from a backyard chicken (GenBank accession number HQ334182), was found in the ceca of four hens (Table 1, hen numbers 4, 10, 11 and 24), but not in livers. Mixed infections by either *T. gallinarum* strain 13/16632 or *T. gallinarum* strain GPO and *Simplicimonas* sp. strain GABC1 were detected in three hens (Table 1, hen numbers 4, 11 and 24).

Wild ducks tested in our survey did not show gross post-mortem lesions. Trichomonad DNA was not detected in their ceca except in one animal which was shown to be infected with the same *Simplicimonas* sp. as the one identified in the hens (99% sequence identity with *Simplicimonas* sp. strain GABC1). Trichomonad DNA was not detected in any of the environmental samples.

## 4. Discussion

In the present case report, an outbreak of granuloma disease likely caused by *T. gallinarum* occurring in 2017 in a flock of free range productive layers in the Netherlands is described. Clinical signs and results of post-mortem examination were similar to those observed in the outbreaks of the year 2013 (Landman et al. 2016), *id est* severe persisting mortality and high within flock incidence of granulomas, especially in livers and ceca. The presence of *T. gallinarum* was confirmed by ISH in all liver and ceca granulomas examined. The ISH *T. gallinarum* is specific as cross reactions with *H. meleagridis* and *Simplicimonas* sp. are highly unlikely considering the probe sequence and its alignment with the 18S rRNA of all three protozoa (Table 3), together with the stringent conditions of ISH.

**Table 3.** Alignment of the *T. gallinarum* ISH antisense probe with partial 18S rRNA sequences of *H. meleagridis*, *T. gallinarum* and *Simplicimonas* sp.

*H. meleagridis* (AF293056)	**TAAATGGATAGCAGAAGTAATTCTTGTGCTAATACATGTTTTTAAAATTTCATTGGAAATTAAATACAATGATAAATT**
*T. gallinarum* (AY245110)	**TAAATGGATAGCAGCAGCAACTCTGGTGCTAATACATGCAATTG---TTTCTCCAGAAGTGAATTATGGAGGAAAAGT**
*T. gallinarum* antisense probe	**GCAACTCTGGTGCTAATACATGCAATTG---TTTCTCCA**
*Simplicimonas* sp. (MK496864)	**TACATGGATAGCAGGAGTAATTCTCGTGCTAATACACGCAATTG---TTTCACCGGATATGAGTTACGGTGGAAAAGT**

Nucleotides different from the antisense probe are indicated on grey background: the antisense probe shows 100% homology with the *T. gallinarum* target sequence, six different nucleotides were found for *Simplicimonas* sp. and 14 for *H. meleagridis*. Accession numbers of the compared sequences are indicated in brackets.

Histomonas isolates show genetic diversity (Lollis et al. 2011). Nonetheless, the sequence targeted by the probe shows no difference between the six isolates, which sequences can be found in GenBank (EU647887.1, EU647886.1, EU647885.1, EU647884.1, AJ920323.1 and AF293056.1). The ISH *H. meleagridis* was always negative, and this result can be considered trustworthy in view of the foregoing.

Although *T. gallinarum* DNA was not always detected by PCR in affected livers, ceca, and cultures thereof, infectious causes of granuloma lesions other than trichomonads were excluded. Detection of *T. gallinarum* in affected organs by DNA techniques and/or culture does not suffice to infer a causal relation between the parasite and the granuloma lesions, as avirulent *T. gallinarum* strains able to induce latent infections, *id est* without lesions exist (Amin et al. 2011). In contrast, ISH was able to show the presence of *T. gallinarum* in close proximity of the granulomas and their absence in non-affected (parts of the) organs (Liebhart et al. 2006; Landman and Van Eck 2018). It is therefore necessary for the definitive diagnosis of *T. gallinarum* granulomatosis.

Tiamulin treatment in a dose of 21 mg per kg body weight per day during five days did not seem to have any effect on the course of the disease.

The *T. gallinarum* strain 13/16632 identified in the present study was genetically identical to that of the 2013 outbreak (Landman et al. 2016) and to that detected in Great Britain by Liebhart et al. (2014) in livers of red-legged partridges (*Alectoris rufa*) associated with fatal typhlohepatitis, and therefore it was likely also virulent. Taking also into account that the rearing farm which was considered to be the source of the 2013 outbreaks and the farm which housed the affected flock in 2017 described in the present study were located in the same wetland area, it is hypothesized that the *T. gallinarum* strain 13/16632 persisted in named area during a long period of time either in the flagellate stage or in the cyst or pseudocyst stage (Friedhoff et al. 1991, Pereira-Neves et al. 2003). This speculation is in agreement with observations made in turkey flocks with severe granulomatosis on a Canadian farm, suggestive of the presence of *T. gallinarum* during a long period of time in a pool of stagnant water containing rotting vegetable matter. The disease occurred during two years in successive flocks. Turkeys preferred to drink from the pool as opposed to clean available water. After the pool was fenced off, granulomatosis did not occur anymore (Trylich et al. 1977). In retrospective, likely the disease was caused by *T. gallinarum* (Landman and Van Eck 2017a).

In the present study, *T. gallinarum* and other trichomonad species were not identified in the environmental samples taken at the premises of concern. In general, the identification of parasites in environmental sources remains complex in relation to different parameters including the quality and quantity of parasitic DNA extracted from these samples that may be insufficient for detection by PCR. *T. gallinarum* was also not identified in the ceca of five wild ducks shot at the affected farm, thus leaving the source of the *T. gallinarum* infection in the laying hens unknown. However, as detailed below, one of the wild ducks was shown to be infected by the same *Simplicimonas* sp. isolate as that identified in layer hens. Therefore, it can be postulated that ducks could represent a reservoir of infection for several trichomonad species including *T. gallinarum* and in this regard, several strains of this species have already been identified in wild ducks (Čepička et al. 2005).

Interestingly and unlike our previous study although conducted in the same geographical area and using the same detection and identification methods (Landman et al. 2016), trichomonad strains and species others than the *T. gallinarum* strain 13/16632 have also been identified in 4 of the layer hens tested as well as in one wild duck. The same *Simplicimonas* sp. was found in both, the ceca and/or cultures from ceca of 4 hens and the ceca of a wild duck. This isolate is related to strain GABC1 (99% sequence identity) previously identified by Lollis et al. (2011) in a backyard chicken (*Gallus gallus domesticus*) in the USA with histomonas-like lesions. In addition, a *T. gallinarum* isolate closely related to the strain GPO (99% sequence identity) was found in cultures of protozoa from livers and ceca of a layer hen. The *T. gallinarum* strain GPO was isolated from *Gallus gallus* in 1999 and later characterized at the DNA level (Čepička et al. 2005). In a recent study, a 5-month-old leucistic Indian peafowl (*Pavo cristatus*) from Southern Brazil presented numerous gross lesions in various organs including nodules in liver and cecum (Michelazzo et al. 2017). The causative agent was identified as *H. meleagridis* strain UEL-1 by these authors since the ITS1-5.8S rRNA-ITS2 sequence obtained from this isolate showed 99% identity with the homologous sequences of the *H. meleagridis* isolates YZ10 and YZ13 deposited in GenBank (Accession numbers KJ863549 and KJ863552, respectively). However, in the phylogenetic study performed by the same authors, the sequences of the isolates UEL-1, YZ10 and YZ13 were shown to be very distant from those of other *H. meleagridis* isolates leading to the most unlikely polyphyly of the genus *Histomonas*. This strongly suggests the misdiagnosis of the isolates UEL-1, YZ10 and YZ13 as *H. meleagridis*, which is further strengthened by the fact that the sequences of these three isolates exhibit 100% identity with that of *T. gallinarum* strain GPO. Moreover, they were closely related to those of various *T. gallinarum* strains in the phylogenetic tree presented by Michelazzo et al. (2017). Consequently, *T. gallinarum* strain GPO identified in the present study in the Netherlands was previously found amongst others in Brazil in a peafowl presenting nodules in liver and ceca.

The severe granuloma disease outbreaks observed in 2013 in productive layer flocks housed on different farms in the Netherlands were shown to be caused only by the *T. gallinarum* strain 13/16632 (Landman et al. 2016; Landman and Van Eck 2018). This trichomonad strain, which was also identified in the present survey on a farm located near the farm that was considered as the source of the 2013 outbreaks, likely represents the main cause of pathological findings found in layer hens described here. However, since additional trichomonad species such as the *T. gallinarum* strain GPO and *Simplicimonas* sp. were also found, the quest of their respective pathogenicity in these animals remains to be clarified.
